# When bullets unmask hidden fire: a rare case of undiagnosed small-vessel vasculitis revealed by dual surgical emergencies

**DOI:** 10.1093/jscr/rjaf496

**Published:** 2025-07-11

**Authors:** Q Carlos Díaz

**Affiliations:** Research Medical Department, Universidad Francisco Marroquin, Guatemala, Guatemala City, Guatemala

**Keywords:** penetrating neck trauma, exploratory laparotomy, small vessel vasculitis, bowel ischemia, dual surgical approach, incidental intraoperative finding

## Abstract

A 36-year-old previously healthy male presented with a gunshot wound to Zone II of the right neck. Surgical exploration revealed no major injuries, and recovery was initially uneventful. However, 48 hr later, the patient developed signs of acute abdomen, prompting an exploratory laparotomy that uncovered 150–180 cm of ischemic small bowel and pale, patchy lesions suggestive of vasculitis. Histopathology confirmed necrotizing vasculitis of small and medium vessels. This case illustrates how an incidental trauma led to the diagnosis of a silent but life-threatening systemic disease. The coexistence of vascular trauma and spontaneous ischemic bowel highlights the importance of considering systemic vasculitides in patients with unexplained abdominal findings, even when presenting with an unrelated surgical emergency.

## Introduction

Vasculitides are a heterogeneous group of disorders characterized by inflammation and necrosis of blood vessel walls, often resulting in tissue ischemia. Small- and medium-vessel vasculitides, including polyarteritis nodosa (PAN) and microscopic polyangiitis (MPA), can involve multiple organs and often go undiagnosed until significant organ damage occurs. Abdominal involvement is seen in ~35%–50% of patients with systemic vasculitis, most commonly manifesting as mesenteric ischemia, bowel perforation, or GI bleeding [1]. However, the presentation is often nonspecific, including mild abdominal discomfort, weight loss, and constitutional symptoms, which may delay diagnosis.

In trauma surgery, cases involving neck exploration and bowel ischemia are frequent but typically unrelated. Zone II neck injuries account for the majority (60%–70%) of penetrating cervical trauma, with vascular injury occurring in about 25% of these cases [2]. Meanwhile, acute mesenteric ischemia (AMI) represents about 1 in 1000 hospital admissions and is associated with high mortality rates, especially when diagnosis is delayed [3]. To our knowledge, there are no prior reports of dual surgical presentations in a single patient, one due to external trauma and the other to an undiagnosed autoimmune pathology.

This case highlights how a penetrating neck injury provided the opportunity to diagnose a clinically silent but pathologically aggressive systemic vasculitis. The early recognition of subtle systemic signs was crucial in guiding the patient toward appropriate medical management postoperatively.

## Case presentation

A 36-year-old male with no significant past medical history and no known drug allergies arrived at the emergency department after sustaining a single gunshot wound to the right lateral neck (Zone II) during an attempted robbery. Remarkably, he was alert and oriented with a Glasgow Coma Score of 15. His vital signs were stable, and he denied any loss of consciousness, dyspnea, or dysphagia. Physical examination showed an entry wound at the level of the thyroid cartilage, with minimal external bleeding and no expanding hematoma or audible bruit.

**Figure 1:**
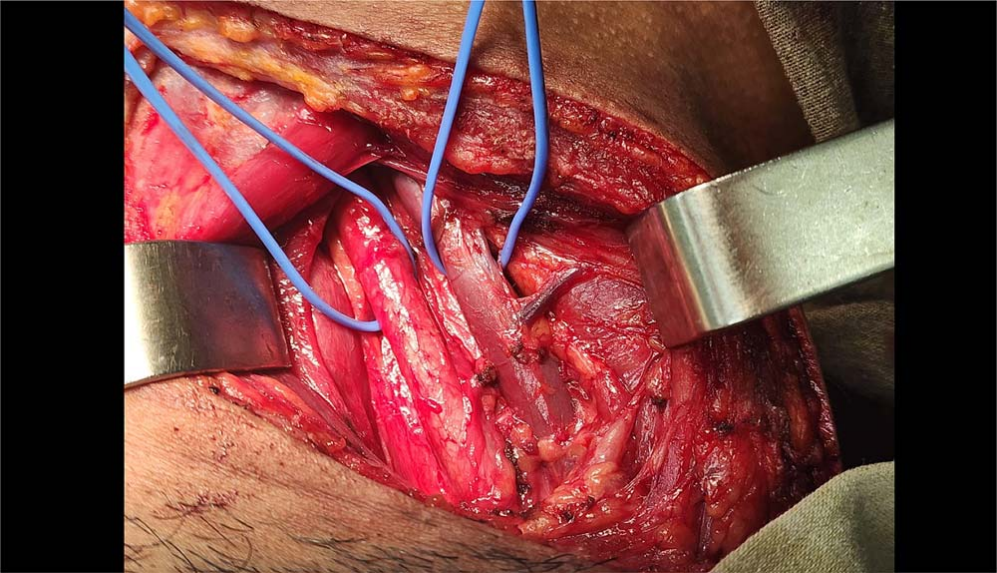
Intraoperative exposure of zone II neck following gunshot injury. This image demonstrates the surgical field during exploration of a penetrating neck wound in zone II on the right side. An oblique cervical incision was made, and the platysma and sternocleidomastoid muscle were dissected to expose the carotid sheath. Key vascular structures, including the common carotid artery and internal jugular vein, were visualized and confirmed intact. No injury to the esophagus, trachea, or vertebral bodies was identified. The operative site is shown prior to layered closure.

Due to platysma violation and potential involvement of major neurovascular structures, the patient was promptly taken to the operating room. A right oblique cervical incision was performed, with dissection through the platysma and sternocleidomastoid muscle to reach the carotid sheath (Fig. 1). No vascular, tracheal, or esophageal injuries were identified. The prevertebral space was also explored, confirming absence of bony or soft tissue damage. Meticulous hemostasis was achieved, and the surgical wound was closed in layers.

Postoperatively, the patient was stable and transferred to the surgical ward. However, by postoperative day two, he began experiencing vague abdominal discomfort, which escalated to severe pain with guarding and rebound tenderness. He developed tachycardia and mild hypotension. Abdominal X-ray revealed air-fluid levels, and contrast-enhanced CT demonstrated small bowel wall thickening and pneumatosis intestinalis, hallmarks of bowel ischemia. An emergent midline exploratory laparotomy was indicated.

**Figure 2:**
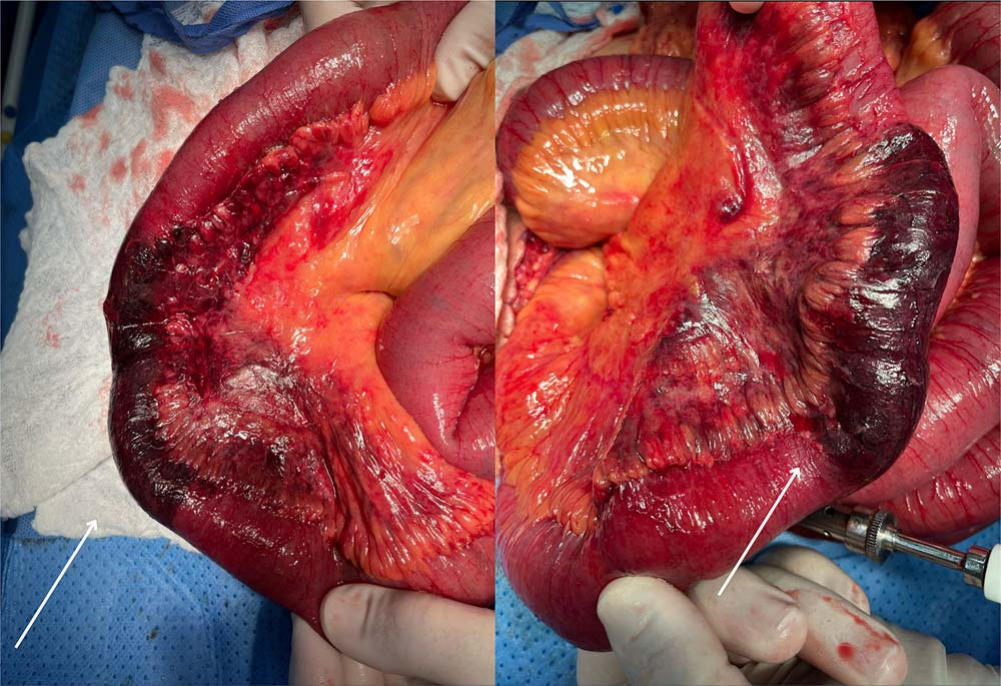
Ischemic small bowel intraoperatively revealed during exploratory laparotomy. Intraoperative view of the small intestine demonstrating extensive ischemic changes involving ~150–180 cm of jejunum and proximal ileum. The affected bowel segments appear dusky with patchy white serosal lesions. The mesentery shows congestion and edema, without evidence of torsion or thrombosis. These findings were consistent with transmural infarction secondary to vasculitis, later confirmed by histopathology. Healthy proximal and distal margins were established prior to resection and anastomosis.

Intraoperative findings revealed ~150–180 cm of distal jejunum and proximal ileum with irreversible ischemic changes, including dusky coloration and patchy white lesions (Fig. 2). The mesentery appeared edematous, with no visible thrombosis or torsion. Additionally, pale, infarcted appendices epiploicae were noted. A segmental bowel resection was performed, followed by a side-to-side stapled anastomosis using a 55 mm GIA device. Mesenteric defects were closed, and the abdomen was irrigated and closed.

Histopathological analysis of the resected bowel confirmed transmural necrosis and a dense lymphocytic infiltrate affecting small and medium vessels, with fibrinoid necrosis features diagnostic of necrotizing vasculitis. Serologic testing returned positive for p-ANCA and ANA (1:160, speckled pattern), alongside elevated inflammatory markers (CRP 35 mg/l, ESR 72 mm/hr). A rheumatology consult confirmed the diagnosis of systemic small-vessel vasculitis, most consistent with MPA. The patient was initiated on pulse corticosteroids and later transitioned to oral immunosuppression with close follow-up.

## Discussion

This case exemplifies the intersection of trauma surgery and autoimmune pathology, highlighting the diagnostic complexity that arises when systemic diseases masquerade as surgical emergencies. The initial presentation of a gunshot wound to the neck was an isolated traumatic event that demanded prompt surgical evaluation but gave no overt clue to an underlying systemic illness [4]. The subsequent development of AMI served as a pivotal clue leading to the discovery of previously undiagnosed necrotizing vasculitis.

The gold standard for diagnosing vasculitis remains histopathologic examination, particularly when supported by serologic markers such as ANCA [5]. In this case, bowel histology provided clear evidence of necrotizing inflammation of the vessel walls. While imaging modalities such as CT angiography and MR angiography can suggest vasculitis through findings like vessel wall thickening or stenosis, they are less sensitive in small-vessel disease and may delay definitive diagnosis if relied upon solely [6].

Surgically, penetrating Zone II neck injuries warrant exploration when platysma is violated, especially with concern for vascular compromise. While endovascular approaches are emerging, open exploration remains the standard in hemodynamically stable patients with suspected multi-structural involvement [7]. For ischemic bowel secondary to vasculitis, surgical resection with primary anastomosis is the preferred approach in stable patients. However, alternative techniques such as temporary ostomies or second-look laparotomies may be indicated in unstable or uncertain scenarios [8].

A novel aspect of this case is the subtlety of early systemic signs, such as unexplained fatigue, intermittent arthralgias, and mild weight loss over the prior month, clues that were initially attributed to post-traumatic stress. In retrospect, these symptoms point toward an indolent vasculitic process. This emphasizes the need for trauma surgeons to remain vigilant for underlying medical conditions in patients whose clinical trajectories deviate from the expected postoperative course.

Moreover, immunosuppressive therapy initiated after surgical stabilization is essential to prevent recurrence. Glucocorticoids remain the first-line treatment, often followed by steroid-sparing agents such as azathioprine, methotrexate, or cyclophosphamide, depending on disease severity [9]. Biologics like rituximab are increasingly used in ANCA-associated vasculitis. In this case, the patient was stabilized postoperatively and started on systemic immunosuppression, avoiding further complications [10].

Lastly, the presence of infarcted appendices epiploicae is an under-recognized finding that further supports a systemic vascular etiology. Their involvement suggests that the vasculitis was diffuse and not localized to a single mesenteric region, underscoring the widespread vascular injury that can accompany seemingly isolated abdominal complaints.

This case highlights the inherent limitations of case reports, including the inability to generalize findings or establish causality. The emergent nature of the presentation restricted a complete autoimmune workup, and long-term follow-up was unavailable. These factors reflect the diagnostic and practical challenges of identifying systemic disease during acute surgical emergencies.

## Conclusion

This case underscores the critical importance of maintaining a broad differential diagnosis in surgical patients, particularly when their clinical course deviates from the expected recovery. A seemingly isolated traumatic event, the gunshot wound unveiled a silent but life-threatening case of small-vessel necrotizing vasculitis. Timely surgical intervention not only treated the acute complications but also allowed for definitive diagnosis via histology. Surgeons must be prepared to recognize when systemic disease mimics or accompanies surgical emergencies. Multidisciplinary collaboration, especially with rheumatology, was key to the patient's favorable outcome. Trauma may demand immediate attention, but underlying systemic pathology can pose the greater, hidden threat, and addressing it can be lifesaving.

## Data Availability

All data supporting this case report are included in the article, with no additional data available due to patient confidentiality.
